# The determinants regulating *Toxoplasma gondii* bradyzoite development

**DOI:** 10.3389/fmicb.2022.1027073

**Published:** 2022-11-11

**Authors:** Ming Pan, Ceng-Ceng Ge, Yi-Min Fan, Qi-Wang Jin, Bang Shen, Si-Yang Huang

**Affiliations:** ^1^Institute of Comparative Medicine, College of Veterinary Medicine, Yangzhou University, Yangzhou, China; ^2^Jiangsu Key Laboratory of Zoonosis, Jiangsu Co-innovation Center for Prevention and Control of Important Animal Infectious Diseases and Zoonoses, Yangzhou, China; ^3^State Key Laboratory of Agricultural Microbiology, College of Veterinary Medicine, Huazhong Agricultural University, Wuhan, China; ^4^Joint International Research Laboratory of Agriculture and Agri-Product Safety, The Ministry of Education of China, Yangzhou University, Yangzhou, China

**Keywords:** *Toxoplasma gondii*, bradyzoite, tachyzoite, cyst, differentiation, immune response, metabolism

## Abstract

*Toxoplasma gondii* is an obligate intracellular zoonotic pathogen capable of infecting almost all cells of warm-blooded vertebrates. In intermediate hosts, this parasite reproduces asexually in two forms, the tachyzoite form during acute infection that proliferates rapidly and the bradyzoite form during chronic infection that grows slowly. Depending on the growth condition, the two forms can interconvert. The conversion of tachyzoites to bradyzoites is critical for *T. gondii* transmission, and the reactivation of persistent bradyzoites in intermediate hosts may lead to symptomatic toxoplasmosis. However, the mechanisms that control bradyzoite differentiation have not been well studied. Here, we review recent advances in the study of bradyzoite biology and stage conversion, aiming to highlight the determinants associated with bradyzoite development and provide insights to design better strategies for controlling toxoplasmosis.

## Introduction

*Toxoplasma gondii* is a cyst-forming obligate intracellular protozoan. Due to its wide host range, *T. gondii* is considered one of the most successful parasitic pathogens (Montoya and Liesenfeld, 2004). In intermediate hosts (almost all warm-blooded vertebrates), this parasite exhibits a multi-stage life cycle through well-designed growth trajectories to promote its infection and spread (Jeffers et al., 2018).

After invading nucleated cells, *T. gondii* proliferates in a rapidly dividing form called tachyzoite, spreading widely in intermediate hosts and causing acute infection (Hakimi et al., 2017). Tachyzoites replicate within parasitophorous vacuole (PV) that serves as a protective niche (Augusto et al., 2020). PV protects *T. gondii* virulent strains from host immune responses in mouse and human cells. However, IFN-γ-dependent cell-mediated immune defense contributes to the control of moderately virulent parasites (Pifer and Yarovinsky, 2011). In an immunocompetent host, tachyzoites eventually differentiate into semi-dormant bradyzoites that are located within tissue cysts, initiating chronic infection in living cells (Jeffers et al., 2018). Within immunosuppressed HIV-infected individuals and other immunocompromised hosts, bradyzoites may reactivate and convert into tachyzoites, resulting in severe zoonotic toxoplasmosis characterized by tissue destruction (Pan et al., 2017; Wang et al., 2017).

Currently, drugs targeting enzymes in the metabolic pathways of *T. gondii* are effective in treating acute toxoplasmosis. Sulfadiazine and pyrimethamine that inhibit dihydropteroate synthase (DHPS) and dihydrofolate reductase (DHFR), respectively, are widely used because of their significant activities against tachyzoites (Alday and Doggett, 2017). During chronic infection, highly active antiretroviral therapy can significantly improve the survival rate of HIV patients (Hoffmann et al., 2007). Drugs such as inhibitors of phenylalanyl-tRNA synthetase (PheRS) and guanabenz alleviate chronic infection in mice (Martynowicz et al., 2019; Radke et al., 2022). However, there is no clinical treatment to eradicate the chronic stage of infection (Alday and Doggett, 2017). On the other hand, despite progress in vaccine development, there is no effective vaccine that completely prevents cyst formation or vertical transmission (Wang et al., 2019).

Bradyzoites pose a potential threat to intermediate hosts due to their ability to persist in living cells. Therefore, the mechanism of bradyzoite differentiation can be an important target for therapeutic intervention. In this review, we review recent advances in bradyzoite biology and bradyzoite development, aiming to highlight the determinants regulating *T. gondii* differentiation and provide a reference for the prevention and control of toxoplasmosis.

## Characteristics of tachyzoite and bradyzoite

### Morphology and ultrastructure

**FIGURE 1:**
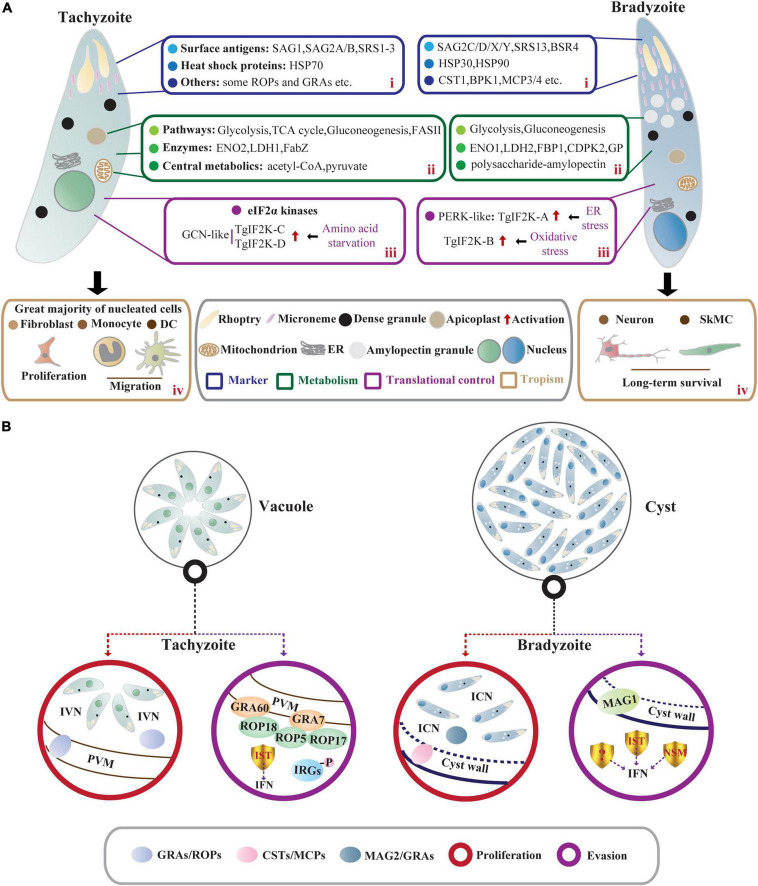
Comparisons of *T. gondii* acute stage and chronic stage in intermediate hosts. **(A)** Characteristics of tachyzoites and bradyzoites. In intermediate hosts, the rapidly dividing tachyzoites and slowly growing bradyzoites differ in many characteristics, including (i) stage-specific markers; (ii) metabolism; (iii) translational control; and (iv) tropism. (i) The stage-specific proteins contain SAGs and HSPs as well as some membrane-associated proteins exported to PV membrane (ROPs and GRAs) and cyst wall (CSTs and MCPs). (ii) Central carbon metabolism (the glycolytic pathway and the tricarboxylic acid cycle) and gluconeogenesis allow intracellular tachyzoites to survive promiscuously. However, bradyzoites lack a functional TCA cycle and respiratory chain but accumulate abundant polysaccharide–AG in the cytoplasm. (iii) The eIF2α phosphorylation regulated by TgIF2K-A and TgIF2K-B leads to the reprogramming of the transcriptome and initiation and maintenance of parasite latency. TgIF2K-C and TgIF2K-D have been reported to protect tachyzoite against amino acid starvation. (iv) The tachyzoites can infect almost all nucleated cells especially fibroblasts, and hijack monocytes and DCs to promote their systemic dissemination. These tachyzoites can spontaneously differentiate into bradyzoites, forming semi-dormant cysts in brain cells (neurons) and muscle cells (SkMCs). **(B)** The proliferation and immune evasion of parasites. In tachyzoite, ROPs and GRAs secreted from rhoptries and dense granules contribute to the formation of PVM and IVN. Meanwhile, IST-dependent suppression of interferon signaling and ROP18 virulence complex (ROP18/ROP5/ROP17/GRA7/GRA60)- dependent phosphorylation of IRGs contribute to parasite evasion from immune-mediated destruction. Bradyzoites within the cyst are encapsulated by the cyst wall (the components include CSTs, MCPs, etc.). The cyst wall is organized into dense and loose layers, and tubules and vesicles are present in an ICN of the cyst matrix, linking bradyzoites together and connecting bradyzoites to the cyst wall. The cyst matrix protein MAG2 interacts with the ICN and GRAs and contributes to the maturation of the cyst matrix. In addition, IST, NSM, and MAG1 act as *T. gondii* immunomodulatory effectors to prevent host cell death and counteract the host immune responses.

Tachyzoite (trophozoite) has a distinct crescent shape and is approximately 2 × 6 μm in size (Sullivan and Jeffers, 2012). Tachyzoites have specific secretory organelles (the rhoptries, micronemes, and dense granules) located at the apical end of the parasites (Kemp et al., 2013; Figure 1A). ROPs and GRAs secreted from rhoptries and dense granules contribute to the formation of PV membrane (PVM), and intravacuolar network (IVN) that connects tachyzoites within the PV and the tachyzoites to the PVM (Kemp et al., 2013; Mercier and Cesbron-Delauw, 2015; Figure 1B). In addition, tachyzoites contain endosymbiotic organelles (the apicoplast and mitochondrion) and general organelles such as endoplasmic reticulum (ER) (Sullivan and Jeffers, 2012; Figure 1A). Tachyzoites replicate in PV by endodyogeny, which requires tightly orchestrated divisions of organelles, including mitochondrion and apicoplast (Verhoef et al., 2021).

Bradyzoites (approximately 1.5 × 7 μm) contain significantly more micronemes than tachyzoites, and the nucleus is located at the posterior end of the parasite (Weiss and Kim, 2000; Figure 1A). High levels of amylopectin granules are accumulated in the cytoplasm of bradyzoites, which may be a long-term energy reserve for the rapid reactivation of bradyzoites under favorable conditions (Uboldi et al., 2015; Figure 1A). The slowly growing bradyzoites within the cyst are encapsulated by a modified PVM called cyst wall (<0.5 μm thick) (Sullivan and Jeffers, 2012). Images of electron microscopy show a filamentous appearance of the cyst wall. Filaments are organized into dense and loose layers that are permeable to small molecules (<10 kDa), and the tubules and vesicles are present in an intracyst network (ICN) of the cyst matrix, linking bradyzoites together and connecting bradyzoites to the cyst wall (Lemgruber et al., 2011; Jeffers et al., 2018; Figure 1B). Studies using fluorescent reporter proteins targeting different subcellular locations and time-lapse video microscopy have shown that developing bradyzoites divide asynchronously through endodyogeny and endopolygeny, with a dynamic view of the reorganization of secretory organelles (dense granules and micronemes), mitochondrial maintenance, and apicoplast mis-segregation. The cysts can develop through free bradyzoite migration or cyst fission (Dzierszinski et al., 2004). However, bradyzoites fail to egress even in response to Ca^2+^ agonists due to reduced Ca^2+^ levels and attenuated Ca^2+^ signaling (Fu et al., 2021). The size of cyst varies with development time, strains, and parasitic sites but is not related to the asynchronous replication of bradyzoites within the cysts (Di Cristina et al., 2008; Watts et al., 2015). In the early stage of bradyzoite development, the cyst is immature and is estimated to be 5 μm in diameter. In comparison, a mature cyst is about 50–150 μm in diameter and contains 1,000–2,000 bradyzoites or more (Sullivan and Jeffers, 2012).

### Stage-specific markers of tachyzoites and bradyzoites

Tachyzoites and bradyzoites differ not only in morphology but also at the molecular levels (Lyons et al., 2002), the differences in their molecular levels are more convincing to distinguish the two stages. Many stage-specific proteins, such as heat shock proteins (HSPs) and surface antigens (SAGs), have been discovered by several approaches using expressed sequence tags (EST), specific monoclonal antibodies, and subtractive libraries (Jeffers et al., 2018).

Heat shock proteins-encoded chaperones are involved in DNA repair by regulating transcription and protein homeostasis (Dubrez et al., 2020), which also engage in tachyzoite proliferation and stress-induced stage conversion of tachyzoites to bradyzoites (Lyons et al., 2002). The expression of TgHSP70 in tachyzoites of virulent strains can be detected in immunocompetent mice but not in immunocompromised mice, suggesting that TgHSP70 is induced by immunological stresses *in vivo* (Lyons and Johnson, 1995). Meanwhile, the infection of *T. gondii* tachyzoites in host cells upregulates expression of host HSP70 (Mitra et al., 2021). 2-Phenylethynesulfonamide (PES), an HSP70 inhibitor that targets host HSP70 and TgHSP70 homolog, attenuates intracellular tachyzoite proliferation *in vitro* (Mitra et al., 2021), suggesting that stress-induced host HSP70 and TgHSP70_*I*_ are closely related to tachyzoite multiplication (Lyons and Johnson, 1995; Mitra et al., 2021; Figure 1A). Screening the bradyzoite cDNA library with a bradyzoite-specific antibody identifies a small HSP homologous gene *hsp30* (also called *bag1*), indicating that *hsp30/bag1* is a specifically expressed gene in bradyzoites (Bohne et al., 1995; Figure 1A). Mapping of *cis*-acting elements in BAG1 promoter identifies that the minimal sequence bound by *T. gondii* transcription factors is required for the expression of *bag1* gene during bradyzoite differentiation in multiple strains (Behnke et al., 2008; Hong et al., 2017). Mice vaccinated with *bag1*-deletion mutants form significantly fewer cysts, implying that BAG1 is not essential for cyst formation but accelerates the process *in vivo* (Zhang et al., 1999). Moreover, the transcription level of parasite *hsp90* is increased under alkaline and heat shock conditions, and this work further confirms the role of TgHSP90 in bradyzoite differentiation through an *hsp90* knockout study (Sun et al., 2017; Figure 1A).

Most SAGs are glycosylphosphatidylinositol-anchored. SAG1/P30, a ligand to bind glycosylated receptors of host cell, is a major surface protein of *T. gondii* tachyzoite involved in its attachment and invasion (Mineo and Kasper, 1994; Figure 1A). Given the immune protection, SAG1-related vaccines based on recombinant protein and DNA are evaluated in BALB/c mice against congenital or acquired toxoplasmosis. The good protective effect makes it possible to develop cocktail vaccines in combination with other immunogenic antigens (Wang et al., 2019; Khorshidvand et al., 2022). Moreover, the dual fluorescent reporter strain EGS driven by the promoter regions of *sag1* and *bag1* can be generalized as a promising tool to elucidate the developmental biology of *T. gondii* or to screen novel active compounds against acute and chronic toxoplasmosis (Portes and De Souza, 2019). According to the EST database for *T. gondii*, SAG-related sequence proteins (SRS1, SRS2, and SRS3) are structurally related to SAG1 and all are confirmed to be expressed only on the surface of tachyzoites (Manger et al., 1998; Figure 1A). Compared to SAG1, bradyzoite surface antigen 4 (BSR4) is chemically and structurally diverse and acts as an interdomain polymorphic linker that facilitates structural adaptation of ligand binding at the bradyzoite stage (Crawford et al., 2009; Figure 1A). Similar but not completely homologous to SAG1, SAG2-related antigens are composed of SAG2A/SAG2B and SAG2C/SAG2D/SAG2X/SAG2Y, of which SAG2A and SAG2B are expressed only in tachyzoites. The specific expression of SAG2C/2D/2X/2Y in bradyzoites is precisely the same as that of SRS9, and it maintains chronic infection in the mouse brain (Lekutis et al., 2000; Lyons et al., 2001; Kim et al., 2007; Saeij et al., 2008; Figure 1A). Through integrated genomic analysis, Lorenzi et al. (2016) have reported more than 100 SAG/SRS protein-coding genes, and some of them are regulated in a stage-specific manner. Collectively, these studies greatly enrich the composition of SAGs and lay the foundation for further research on the functions of SAGs in tachyzoites or bradyzoites.

### Cyst wall- and cyst matrix-associated proteins

**FIGURE 2:**
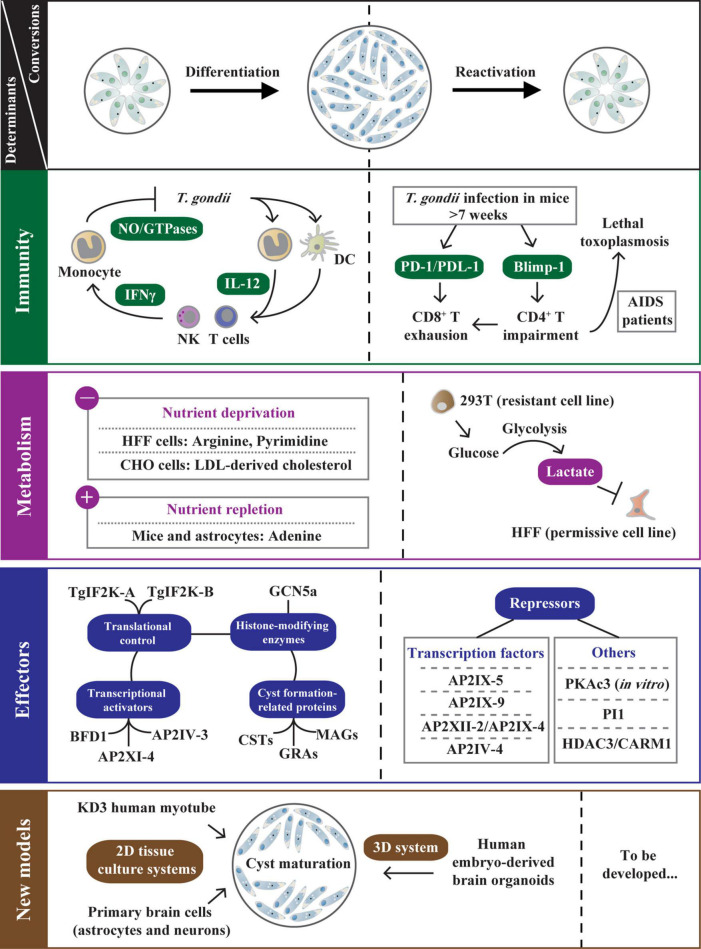
The determinants regulating bradyzoite development and reactivation. After *T. gondii* infection, host microenvironment adjustments (immune response and metabolite supply) are associated with tachyzoite-bradyzoite interconversion. The IL-12/IFN-γ pathway is an effective strategy primarily used to control *T. gondii* infection in immunocompetent hosts. Despite this, some tachyzoites escape immune destruction and differentiate into bradyzoites. In chronically infected mice (weeks postinfection >7) or *T. gondii*-infected AIDS patients, the host CD4^+^ T cell impairment that is regulated by Blimp-1 enhances PD-1/PDL-1-mediated CD8^+^ T cell exhaustion, resulting in bradyzoite reactivation. The host metabolites also directly impact *T. gondii* differentiation under nutrient-deprived conditions (deprivation of arginine, pyrimidine, etc.) or nutrient-replete conditions (exogenous addition of adenosine and lactate). In addition, *T. gondii* relies on various strategies to optimize its persistence, including the transcriptional activation or repression by TgAP2 factors and other effectors (PKAc3, PI1, etc.), the translational control *via* TgIF2K-A/TgIF2K-B-dependent eIF2α phosphorylation, GCN5a-mediated post-translational modifications (PTMs), and the biogenesis of the cyst wall and cyst matrix. Moreover, the development of 2D tissue culture systems with human myotubes and primary brain cells promote cyst maturation, and emerging 3D system with human brain organoids can present the natural cellular structure of *T. gondii* life cycle stages. These new *in vitro* models help elucidate the mechanisms of *T. gondii* stage conversion. The left side of the dotted line represents the determinants regulating bradyzoite differentiation. On the other hand, the factors contributing to bradyzoite reactivation are documented.

The cyst wall glycoprotein CST1 contains *N*-acetylgalactosamine (GalNAc) sugar residues in a mucin-like domain that binds *Dolichos biflorus* agglutinin/lectin (DBA) (Zhang et al., 2001; Figure 1A). This glycoprotein co-localizes with succinylated wheat germ agglutinin (s-WGA), a lectin that recognizes *N*-acetylglucosamine (GlcNAc)-decorated cyst structures (Guevara et al., 2020a). The mucin domain of CST1 is highly *O*-GalNAc glycosylated and modified by the enzymes called polypeptide *N*-acetylgalactosaminyltransferases (ppGalNAc-Ts), which are required for the formation of a mechanically stress-resistant cyst wall (Tomita et al., 2017). One of the isoforms, ppGalNAc-T2, is required for initial glycosylation, while the other, ppGalNAc-T3, is responsible for sequential glycosylation. Lack of *O*-GalNAc glycosylation in ppGalNAc-T2 and -T3 mutants results in the formation of fragile brain cysts, consistent with CST1 deletion, indicating that glycosylated CST1 is necessary to confer structural rigidity on cysts (Tomita et al., 2013, 2017). Subsequently, using microarray approach and BioID technique as well as a proteomic analysis, the cyst wall components are expandable to include BPK1, MCP3/MCP4, SRS13, and CST2-10 (Buchholz et al., 2011; Tomita et al., 2018; Tu et al., 2019, 2020b; Figure 1A). Among them, CST2 is positively correlated with parasite virulence and cyst burden (Tu et al., 2019). Like CST1, another glycoprotein proteophosphoglycan (TgPPG1) is upregulated in bradyzoites and enhances cyst wall formation (Craver et al., 2010). In addition to proteins involved in cyst wall formation, the cyst matrix antigen MAG1 is expressed in both tachyzoites and bradyzoites. It is involved in parasite growth and cystogenesis, so MAG1 is a promising chimeric antigen for the serodiagnosis of toxoplasmosis (Drapala et al., 2015; Tu et al., 2020a; Tomita et al., 2021b). MAG2 (also known as brain cyst load-associated antigen, BCLA) localizes within the cyst matrix (Dard et al., 2021), interacts with ICN, and may coordinate with previously reported PVM-localized GRAs and IVN-associated GRAs to regulate the maturation of the cyst matrix and cyst wall (Guevara et al., 2019, 2020b; Tu et al., 2020a; Figure 2). Serological studies based on these characteristics of MAG2/BCLA confirm that it can be used as a biomarker to detect chronic infection in humans (Dard et al., 2021). Additionally, the nucleotide-sugar transporter TgNST1, the abundant bradyzoite antigen SRS9, and the scramblase TgATG9 play a critical role in the efficient long-term persistence of the cyst (Kim et al., 2007; Caffaro et al., 2013; Smith et al., 2021).

### Metabolism

Intracellular tachyzoites mainly utilize host-derived glucose as a carbon source and then catabolize glucose for biogenesis *via* different metabolic pathways (MacRae et al., 2012; Figure 1A). In the glycolytic pathways, the highly active enolase 2 (ENO2) and pyruvate kinase (PYK) of tachyzoite catalyze phosphoglycerate to pyruvate and generates ATP (Tomavo, 2001; Figure 1A). Pyruvate is the core of the carbon metabolism of *T. gondii* and produces acetyl-CoA that enters the TCA cycle. A small fraction of pyruvate is converted to lactate by lactate dehydrogenase (LDH) (Oppenheim et al., 2014; Figure 1A). *Ldh1* is a developmental regulatory gene expressed only in tachyzoites, and deletion of *ldh1* reduces acute parasite virulence and cyst burdens in chronically infected mice (Abdelbaset et al., 2017; Figure 1A). In the absence of glucose, tachyzoites can fuel the TCA cycle with glutamine-derived carbon and then enter gluconeogenesis through phosphoenolpyruvate carboxykinase (PEPCK) to replenish glycolytic intermediates (MacRae et al., 2012; Nitzsche et al., 2017; Xia et al., 2019). These studies reveal that *T. gondii* tachyzoites are metabolically flexible, allowing them to survive promiscuously. Given the fatty acid (FA) synthesis, FASI and FASII pathways are performed in the cytosol and apicoplast of *T. gondii*, respectively (Ramakrishnan et al., 2015). Parasites lacking FASI do not exhibit any damage (Tymoshenko et al., 2015). However, *de novo* FASII disruption results in the formation of more “tethered” daughter cells and prevents cytokinesis of tachyzoites as revealed by morphological analyses (Martins-Duarte et al., 2016). Tachyzoites lacking FabZ (an enoyl-CoA hydratase catalyze the third step of the FASII pathway) display severe lytic cycle defects, indicating that FASII plays an essential role in the survival of tachyzoites (Krishnan et al., 2020a; Figure 1A). To assist tachyzoites in obtaining vitamin and cofactor, thiamine diphosphokinase (TPK) converts scavenged Vitamin B_1_ to a cofactor thiamine pyrophosphate (TPP), and TPK is critical for *in vitro* tachyzoite survival due to its high negative fitness scores based on the genome-wide CRISPR screen in *T. gondii* (Sidik et al., 2016; Krishnan et al., 2020b; Lunghi et al., 2022). In addition, pantothenate kinases (PanK1/PanK2) that catalyze the phosphorylation of pantothenate (Vitamin B_5_) to initiate CoA biosynthesis are shown to be essential for tachyzoite growth (Sidik et al., 2016; Krishnan et al., 2020b; Lunghi et al., 2022).

In bradyzoites, succinate dehydrogenase (SDH, one of the markers of mitochondrial function) activity cannot be detected, indicating that bradyzoites lack a functional TCA cycle and respiratory chain (Fleige et al., 2008). However, the metabolic enzymes of the glycolysis and gluconeogenesis pathways are expressed with higher catalytic activities to meet the carbon demand of bradyzoites (Kibe et al., 2005; Fleige et al., 2008; Xia et al., 2018; Figure 1A). In the glycolytic pathways of bradyzoites, two upstream repression sites are found in the *eno2* promoter, while the promoter activity of *eno1* is highly induced (Kibe et al., 2005; Figure 1A). Disruption of *eno1* results in changes in *T. gondii* gene transcription and decreases brain cysts in chronically infected mice, indicating that ENO1 functions as a transcriptional regulator in *T. gondii* differentiation (Mouveaux et al., 2014). LDH2 that has higher catalytic activity is also highly expressed in bradyzoites and plays a secondary role in cyst formation (Xia et al., 2018; Figure 1A). On the other hand, the gluconeogenesis enzyme FBP1 and two glucose phosphate-mutase (GPM) isoforms, which appear to produce glucose-6-phosphate for amylopectin synthesis, are upregulated in bradyzoites (Fleige et al., 2008; Figure 1A). Bradyzoites accumulate abundant polysaccharide-amylopectin granules (AG) in the cytoplasm as a long-term energy reserve for their rapid reactivation under favorable conditions (Dubey et al., 1998; Uboldi et al., 2015; Figure 1A). Through bioinformatic analyses, many enzymes involved in amylopectin biosynthesis (UDP-glucose pyrophosphorylase, amylopectin synthase, branching enzymes, etc.) and amylopectin degradation (debranching enzymes, α-amylase, dikinase, etc.) have been confirmed (Coppin et al., 2005). However, there is no clear indication that an enzyme plays a crucial role in starch metabolism in bradyzoites. A study of Ca^2+^-dependent protein kinase CDPK2 in *T. gondii* reveals that CBM20 domain of TgCDPK2 facilitates amylopectin binding. In contrast, deletion of TgCDPK2 or the functional CBM20 domain leads to aberrant and massive amylopectin accumulation in bradyzoites, which eventually leads to parasite death and elimination of cyst formation *in vivo* (Uboldi et al., 2015; Figure 1A). Using ^13^C-U-glucose labeling, amylopectin abundance is significantly elevated in Δ*cdpk2* parasites, indicating that CDPK2 regulates amylopectin synthesis and degradation by increasing glycolytic flux. Quantitative proteomics and substrate capture techniques identify that CDPK2 can directly phosphorylate pyruvate phosphate dikinase (PPDK) (Uboldi et al., 2015). Further study discovers glycogen phosphorylase (GP) is involved in storing amylopectin (Sugi et al., 2017; Figure 1A). Site-directed mutagenesis from Ser25 of GP to Ala or a stop codon leads to amylopectin accumulation. At the same time, the phosphorylation-mimetic mutant (Ser25 to Glu) results in amylopectin deficiency, but all these mutants produce significantly decreased brain cysts in infected mice (Sugi et al., 2017). The study reveals that GP, like CDPK2, is involved in the storage and utilization of amylopectin, and the interaction between CDPK2 and GP needs further investigation. Moreover, a recent study reveals that the pantothenate (Pan) biosynthesis pathway is crucial for *T. gondii* chronic infection *in vivo*, and this pathway is dispensable in tachyzoites or bradyzoites that are differentiated *in vitro*, this finding provides a target for intervention against *T. gondii* bradyzoites (Lunghi et al., 2022).

### Tropism

*Toxoplasma gondii* tachyzoites can infect almost all nucleated cells of warm-blooded vertebrates (Smith et al., 2020). Recent findings indicate that tachyzoites induce the migration of monocytes and dendritic cells (DCs), thereby promoting the systemic dissemination of parasites (Drewry et al., 2019; Sangare et al., 2019; Figure 1A). After reaching terminally differentiated cells, including muscle cells and brain cells, tachyzoites spontaneously differentiate into bradyzoites, showing a prominent tissue tropism (Matta et al., 2021; Figure 1A).

When C2C12 mouse skeletal muscle cells (SkMCs) are infected with *T. gondii*, the cyst is preferentially triggered in differentiated syncytial myotubes rather than myoblasts (Swierzy and Luder, 2015). The bradyzoite formation is associated with upregulation of the host negative cell cycle regulator Tspyl2 expression and ROS levels (Swierzy and Luder, 2015; Rahman et al., 2021). Overexpression of the ortholog of Tspyl2, cell division autoantigen-1 (CDA1), also promotes *T. gondii* stage conversion in human fibroblasts (White et al., 2014). These findings provide evidence for determining the differentiation of *T. gondii* bradyzoites in muscle cells.

Compared to SkMCs, the presence of a highly impermeable blood–brain barrier (BBB) is a protective niche that restricts the entry of pathogens into the central nervous system (CNS). However, *T. gondii* can cross the BBB and invade various cell types, including neurons and astrocytes, as well as microglia (Matta et al., 2021). Analysis of host–parasite interactions *in vivo* using *T. gondii* expressing Cre recombinase reveals that neurons are the primary host cells for cysts (Wohlfert et al., 2017). However, a recent study shows that a subset of neurons can respond to IFN-γ and clear intracellular parasites (Chandrasekaran et al., 2022). Thus, more studies are needed to update the research progress of the cyst-neuron interactions from metabolic or immunological analysis to unveil unknown niche that neurons provide for bradyzoite development and parasite clearance.

## Sensing mechanisms of the host are correlated with parasite control and bradyzoite development

After primary *T. gondii* infection, several host-derived anti-parasitic mechanisms have been developed to limit parasite growth. The work focuses on how host microenvironment adjustments control *T. gondii* infection. This section lists host-sensing tools related to parasite control and bradyzoite differentiation/activation, including immune response and metabolite supply.

### Immune response

The γ-interferon (IFN-γ) pathway is an effective strategy primarily used to control *T. gondii* infection in different hosts (Tomita et al., 2021a; Figure 2). In the early stage of *T. gondii* infection in mice, the parasite profilin is initially recognized only by murine Toll-like receptors on DCs which are absent or non-functional in humans. This interaction is essential for host production of the pro-inflammatory cytokine interleukin-12 (IL-12) (Hunter and Sibley, 2012; Fisch et al., 2019). The IL-12 activates NK cells and T cells to release IFN-γ, resulting in the activation of a variety of anti-parasitic activities, specifically upregulating immunity-related GTPases (IRGs) (Hunter and Sibley, 2012; Poncet et al., 2019; Khan and Moretto, 2022; Figure 2). Unlike mice, humans depend on various other innate mechanisms and effectors (NO) to respond to *T. gondii*. However, the IFN-γ is still the major mediator in controlling *T. gondii* infection in humans (Fisch et al., 2019; Figure 2). The hierarchical innate and cell-mediated immune responses in mice and humans play a critical role in the early control and clearance of intracellular tachyzoites.

After infection with *T. gondii* in C2C12 SkMCs, IFN-γ stimulation significantly inhibits parasite growth *via* inducing IRGs and NO, suggesting that IFN-γ also plays a pivotal role against *T. gondii* in skeletal cells (Takacs et al., 2012; Wohlfert et al., 2017). However, whether other muscle cells are involved in parasite control remains unclear. In addition, tachyzoites spontaneously differentiate into bradyzoites in SkMCs (Swierzy and Luder, 2015), and the immunomodulatory role of SkMCs on bradyzoites is also unknown. Further extensive research into how persistent immune surveillance works will help establish a relatively complete network of *T. gondii–*host interactions in infected SkMCs.

In the early stage of cerebral toxoplasmosis, neutrophil granulocytes recruited from the periphery can infiltrate infection sites in the CNS (Biswas et al., 2017). The study identifies those two subsets of neutrophils in the brain, CD62-L^low^ and CD62-L^high^, produce different levels of IFN-γ to mediate host resistance to *T. gondii* (Biswas et al., 2017). Reduced IFN-γ production and increased parasite burden are detected in the brains of infected mouse by treatment with anti-Ly6G, a neutralizing antibody for neutrophils, suggesting that the neutrophils are critical for parasite control (Biswas et al., 2017; Debierre-Grockiego et al., 2020). Astrocytes in the brain are essential effector cells required for *T. gondii* control. After *T. gondii* infection and IFN-γ stimulation, astrocytes induce the STAT1 phosphorylation and recruit the p47 GTPase IGTP to limit parasite growth (Hidano et al., 2016). Astrocytes lacking STAT1 are unable to restrict parasite replication and thereby allow the formation of increased numbers of cysts in astrocytes and bystander cells (Hidano et al., 2016). This indicates that IFN-γ/STAT1 signaling in astrocytes is involved in parasite control and cyst formation (Hidano et al., 2016; Wohlfert et al., 2017). In neurons, neuronal MHC I presentation of the tachyzoite antigen GRA6 by infected neurons induces CD8^+^ T cell-specific responses, resulting in robust control of brain-persisting cysts during the chronic phase (Salvioni et al., 2019). A recent study has found that a portion of murine neurons can clear intracellular parasites in an IFN-γ-IRG-dependent manner both *in vitro* and *in vivo*. IFN-γ stimulation also results in parasite resistance in human neurons (Chandrasekaran et al., 2022). Furthermore, CD40 signaling has also been associated with decreasing cyst emergence in mouse neuroblastoma cells (Doherty et al., 2022).

*Toxoplasma gondii* has evolved sophisticated strategies to bypass the host immune system despite host stress. In tachyzoites, TgIST-dependent suppression of interferon (type I and type II) signaling and ROP18 virulence complex (ROP5/ROP17/ROP18/GRA7/GRA60)-dependent phosphorylation of IRGs contribute to parasite evasion from immune-mediated destruction, resulting in the conversion of tachyzoites to bradyzoites (Gay et al., 2016; Hakimi et al., 2017; Matta et al., 2019; Nyonda et al., 2021; Figure 1B). During the bradyzoite stage of chronic infection, the export of IST into the host nucleus also suppresses the IFN-γ pathway, which plays a vital role in protecting parasites from IFNγ-mediated cell death (Seizova et al., 2022; Figure 1B). Meanwhile, another secreted effector, TgNSM, targets the host’s NCoR/SMRT repressor complex and blocks IFN-induced necroptosis together with TgIST (Rosenberg and Sibley, 2021). At the same time, the cyst matrix protein MAG1 inhibits IL-1β secretion in *T. gondii*-infected BMDM and dampens inflammasome activation (Tomita et al., 2021b; Figure 1B). Thus, a good balance between host anti-parasitic immunity and *T. gondii* antagonism of host immunity is achieved in chronic infection.

In immunocompromised hosts, bradyzoites can dedifferentiate into tachyzoites, leading to a reactivated infection and a potentially fatal outcome (Jeffers et al., 2018). A typical case is that infection of *T. gondii* in AIDS patients can lead to lethal toxoplasmic encephalitis (TE). Pathogenesis is associated with host CD4^+^ T cell impairment and TgROP18-mediated phosphorylation (An et al., 2018; Kupz et al., 2020; Figure 2). Similar reactivation is found in chronically infected mice (weeks postinfection >7), mediated by systemic CD8^+^ T cell depletion (Bhadra et al., 2011; Figure 2). The inhibitory receptor PD-1 and its ligand PDL-1 characterize this dysfunction, and PD-1-PDL-1 blockade reinvigorates CD8^+^ T cell response and further controls toxoplasmosis relapse (Bhadra et al., 2011; Figure 2). Later, a finding demonstrates that CD4^+^ T cells are similarly depleted, even more so than CD8^+^ T cells during chronic toxoplasmosis (Hwang et al., 2016). Unmistakable evidence is that the CD4^+^ T cell-intrinsic deletion of the transcription factor Blimp-1 (a critical regulator for CD4^+^ T cell exhaustion) reverses CD8^+^ T cell dysfunction, indicating that CD4^+^ T cells help to reduce CD8^+^ T cell exhaustion and that both are considered to be critical for improving parasite control to prevent fatal TE in infected mice (Hwang et al., 2016; Figure 2). Studies in AIDS patients and chronically infected mice reveal that disruption of the immune balance between parasite and host leads to the recrudescence of toxoplasmosis.

### Metabolite supply

Due to the wide range of hosts, *T. gondii* is exposed to different host environments, and the parasite can be metabolically flexible to adapt to environmental and physiological changes (Walsh et al., 2022). Evidence for this metabolic flexibility is that tachyzoites and bradyzoites require significantly different central metabolites for intracellular survival. On the other hand, host metabolites directly affect *T. gondii* differentiation under nutrient-deprived or nutrient-replete *in vitro* conditions (Kloehn et al., 2020; Figure 2).

*Toxoplasma gondii* is an arginine auxotroph due to a lack of enzyme for *de novo* arginine biosynthesis (Fox et al., 2004). Arginine starvation blocks the replication of tachyzoites and triggers cyst formation. In contrast, the addition of exogenous arginine or citrulline rescues the growth defect and induces the growth of tachyzoites *in vitro* (Fox et al., 2004; Figure 2). Similar to arginine, *T. gondii* is auxotrophic for low-density lipoproteins (LDL)-derived cholesterol. Tachyzoites increase LDL uptake in CHO cells, whereas deprivation of LDL-derived cholesterol induces bradyzoite conversion (Ihara and Nishikawa, 2014; Figure 2). The *de novo* pyrimidine biosynthesis pathway in *T. gondii* is catalyzed by CPSII, which requires CO_2_ as a substrate for the composite of the pyrimidine ring (Bohne and Roos, 1997; Fox and Bzik, 2002). In the pyrimidine salvage pathway, uracil phosphoribosyl-transferase (UPRT) is easily disrupted, and parasites lacking UPRT exhibit a reduced intracellular replication rate and an increased bradyzoite induction rate under the ambient CO_2_ (0.03%) condition, indicating that pyrimidine deprivation (breaking *de novo* synthesis and salvage pathways) triggers bradyzoite differentiation (Cerutti et al., 2020; Figure 2). Additionally, the conversion of tachyzoites to bradyzoites readily occurs in permissive human foreskin fibroblast (HFF) and Vero cells but not in resistant NIH3T3 and 293T cells *in vitro*, indicating that different cell types differ dramatically in the ability to favor differentiation to bradyzoites (Weilhammer et al., 2012). Lactate is identified as an inhibitory component of 293T supernatants by mass spectrometry and biochemical analysis. High glucose-induced glycolysis disrupts the phenotype of HFF cells, rendering them unable to convert to bradyzoites (Weilhammer et al., 2012; Figure 2).

*In vivo*, host metabolites also control *T. gondii* differentiation. Extracellular adenosine in the mammalian cell is generated by the dephosphorylation of adenosine monophosphate (AMP) *via* a GPI-anchored cell surface glycoprotein-CD73, which is highly expressed on CNS-resident cells (Mahamed et al., 2012). When challenged with *T. gondii* cysts, CD73-deficient mice exhibit reduced bradyzoite differentiation and cyst burden in brain tissue. In stress-induced CD73^–/–^ astrocytes, exogenous adenosine can rescue cyst formation, indicating that host CD73 expression and adenosine production promote *T. gondii* bradyzoite differentiation and cyst formation (Mahamed et al., 2012; Figure 2). Collectively, these findings provide sufficient evidence that different metabolites provided by the host have a significant effect on *T. gondii* stage conversion.

## *Toxoplasma gondii* regulates the self-cellular environment to optimize its persistence

*Toxoplasma gondii* relies on various effectors to promote its growth and development in response to host stress. In addition to immune modulators described above, some *T. gondii* cyst formation-related activators and inhibitors directly affect the regulation of the self-cellular environment, which contributes to *T. gondii* differentiation and optimizes its persistence.

### Transcription activators and repressors

Like the Apetala 2 (AP2) factors found in plants, Apicomplexa express several plant-like proteins called ApiAP2 factors. These ApiAP2 carry DNA-binding domains that cooperate with histone-modifying machinery (acetylation or deacetylation) (Augusto et al., 2020). *T. gondii* currently contains nearly 70 ApiAP2 (Waldman et al., 2020), but only a few have been studied.

AP2XI-4 is a nuclear factor identified early in *T. gondii* and thus exhibits the ability to sequence-specific DNA-binding to regulate gene promoter activities (Walker et al., 2013). This study confirms that AP2XI-4 is involved in tachyzoite–bradyzoite interconversion *in vitro via* bradyzoite gene expression regulation through gene knockout of both RH and Pru strains. More importantly, the *ap2xi-4* transcript level is higher in chronically infected mice than that in tachyzoites, and a significantly decreased cyst burden is detected in mice infected with AP2XI-4 knockout strains. Therefore, this work reveals that AP2XI-4 is used as a regulator to control bradyzoite gene expression (Walker et al., 2013; Figure 2). Since then, other transcription activators have been studied. Under alkaline stress-induced conditions (>48 h), AP2IV-3 is induced and regulates *bag1* transcription to enhance tissue cyst formation (Hong et al., 2017; Figure 2). Disruption of *ap2iv-3* alters the ability of parasites to form cysts, while overexpression of AP2IV-3 reverses the phenotype (Hong et al., 2017). Interestingly, a recent study finds that AP2IV-3 can be regulated by a transcriptional repressor-microrchidia (TgMORC) during the sexual stage, indicating that AP2IV-3 plays multiple roles in the life cycle of *T. gondii* (Farhat et al., 2020). Through Cas9-mediated genetic screening and single-cell profiling, BFD1 is identified as a master regulator contributing to chronic-stage *T. gondii* differentiation (Waldman et al., 2020). This study reflects that loss of BFD1 blocks the parasite differentiation regardless of stress and that BFD1 knockout mutants cannot form cysts in mice. In addition, BFD1 binds to the transcriptional start sites of bradyzoite genes, including *bag1*, *ldh2*, and *eno1*, which is required to initiate differentiation (Waldman et al., 2020; Figure 2). Therefore, BFD1 has become a breakthrough for elucidating the molecular mechanisms of bradyzoite differentiation in *T. gondii*. The tachyzoite transcription factor AP2IX-1 is identified by single-cell RNA sequencing (Xue et al., 2020). Analysis of a single SAG1^–^ outlier cell reveals that AP2IX-1 controls the switch from SAG1 to rare SRS, contributing to antigen expression associated with the acute stage (Xue et al., 2020). Since some SRS are also explicitly expressed in bradyzoites, the role of AP2IX-1 at this developmental stage needs to be followed.

In contrast to the transcriptional activation of bradyzoite differentiation, an opposite mechanism limits the development of *T. gondii* to cysts. AP2IX-9 can be induced by alkaline pH, binding to the *cis*-regulatory elements of bradyzoite nucleoside triphosphatase (B-NTPase) and *bag1* promoters to inhibit the bradyzoite transcriptome in competition with transcription activator AP2IV-3 (Radke et al., 2013; Jeffers et al., 2018). Conditional overexpression of AP2IX-9 inhibits tissue cyst formation, whereas lack of *AP2IX-9* gene promotes developmental pathway, suggesting that AP2IX-9 acts as an inhibitor of bradyzoite development (Radke et al., 2013; Figure 2). The S-phase cell cycle regulator AP2XII-2 is associated with AP2IX-4, another regulator of bradyzoite gene expression (Srivastava et al., 2020). Depleting AP2XII-2 results in cell cycle delay, further increasing the frequency of latent cyst formation *in vitro*, indicating that AP2XII-2 completes cell cycle progression and inhibits tissue cyst formation with AP2IX-4 (Huang et al., 2017; Srivastava et al., 2020; Figure 2). AP2IV-4 inhibits a subset of bradyzoite-specific proteins required for *T. gondii* to establish a chronic disease during acute infection (Radke et al., 2018). Notably, a key transcriptional regulator, TgAP2IX-5, is confirmed to control the activation of asexual cell cycle division and can directly bind the promoters of AP2IV-4 and AP2XII-2, thereby promoting the inhibition of the differentiation pathway in *T. gondii* (Khelifa et al., 2021; Wang et al., 2021; Figure 2). However, the molecular mechanism of AP2IX-5 in elastic regulation of division and differentiation remains unclear. In addition, the deletion of serine protease inhibitor 1 (TgPI1) and protein kinase A catalytic subunit 3 (TgPKAc3) can increase bradyzoite formation *in vitro* (Pszenny et al., 2012; Sugi et al., 2016; Radke et al., 2018; Figure 2). Interestingly, *pi1*-knockout mutant increases parasite burden in infected mice, but ablation of PKAc3 reduces the cyst number *in vivo*, indicating that PKAc3 acts versatile roles in bradyzoite development and maintenance (Pszenny et al., 2012; Sugi et al., 2016). Although there is an opposite way of regulating bradyzoite development, these suppressors are essential in chronic toxoplasmosis because turning off bradyzoite antigen expression is just as important as turning it on (Wohlfert et al., 2017).

### Protein kinases that function in translational control

As for translational control, phosphorylation of the alpha subunit of eukaryotic initiation factor 2 (eIF2α) is associated with stress-induced response in eukaryotic cells for selectively enhancing translation of relevant mRNAs and downregulating global protein expression (Zhang et al., 2013). In *T. gondii*, eIF2α phosphorylation leads to the reprogramming of the transcriptome and initiation and maintenance of parasite latency (Holmes et al., 2017). The eIF2α phosphorylation is mediated by different kinases activated by various cellular stresses. TgIF2K-A is a PERK-like eIF2 kinase that localizes in the ER and binds to the chaperone BiP (Narasimhan et al., 2008). ER stress condition induces the release of BiP from TgIF2K-A, resulting in TgIF2K-A activation and eIF2α phosphorylation. The translational control triggers the expression of bradyzoite-specific genes, which causes cyst development (Narasimhan et al., 2008; Figures 1A, 2). Compared with TgIF2K-A, TgIF2K-B appears to be activated in response to oxidative stress (Holmes et al., 2017; Figure 1A). A recent report finds that deletion of TgIF2K-B disrupts the antioxidant response. This mutant enhances parasite replication and virulence *in vivo* but reduces the ability to transform into tissue cysts. The result suggests that TgIF2K-B is a sensor protein essential for maintaining an oxidative balance of *T. gondii*, which is critical for parasites to establish latent infection (Augusto et al., 2021; Figures 1A, 2). Two dispensable GCN-like eIF2 kinases, TgIF2K-C and TgIF2K-D, are also reported to protect parasites from amino acid starvation (Holmes et al., 2017; Figure 1A). TgIF2K-C increases the viability of intracellular tachyzoites under glutamine starvation conditions, while TgIF2K-D enables extracellular tachyzoites to overcome environmental stress *via* eIF2α phosphorylation (Holmes et al., 2017), but the role of these kinases in bradyzoites remains unknown.

### Histone-modifying enzymes

In addition to their independent effects, many AP2 factors work synergistically with histone-modifying enzymes (Kim, 2018). Due to the dramatic increase in proteome diversity and complexity, post-translational modifications (PTMs) are recognized as critical processes in the biology and pathogenesis of apicomplexan parasites (Yakubu et al., 2018). A classic mode of *T. gondii* PTM is histone acetylation, which is catalyzed by two GCN5-family lysine acetyltransferases (TgGCN5a and TgGCN5b). GCN5b cannot be knocked out, suggesting that it is essential for *T. gondii* viability (Wang et al., 2014). Induced accumulation of mutated GCN5b protein leads to decreased histone H3 acetylation and tachyzoite growth arrest, and GCN5b simultaneously interacts with different pairs of AP2 factors (AP2IX-7/AP2X-8 and AP2XII-4/AP2VIIa-5), indicating that GCN5b is involved in gene regulation during tachyzoite replication (Wang et al., 2014; Harris et al., 2019). Compared with GCN5b, GCN5a is up-regulated in response to alkaline stress to regulate gene expression (Naguleswaran et al., 2010). Under the same condition, parasites lacking GCN5a fail to up-regulate *bag1* and *ldh2* and show defects in stress recovery (Naguleswaran et al., 2010; Figure 2). On the other hand, the histone deacetylase HDAC3, located only in the parasite nucleus, is recruited to the promoter regions of bradyzoite-specific genes, which contributes to gene regulation *via* deacetylation of histone tails (Saksouk et al., 2005). Compound FR235222 explicitly inhibits HDAC3 and induces parasite hyper-acetylation and bradyzoite differentiation *in vitro* (Bougdour et al., 2009; Maubon et al., 2010; Figure 2).

Additionally, coactivator-associated arginine methyltransferase 1 (TgCARM1) is recruited to target H3 [R17] along with GCN5, which perfectly targets H3 [K18] (Saksouk et al., 2005). AMI-1 (an arginine *N*-methyltransferase activity inhibitor) inhibits TgCARM1 activity and induces cyst formation *in vivo* (Saksouk et al., 2005; Figure 2). Protein arginine methyltransferase 1 (TgPRMT1) is involved in monomethyl arginine (MMA) of *T. gondii* (Yakubu et al., 2017). Through proteomic analysis of MMA proteins, substrates of TgPRMT1, including RNA-binding proteins, AP2 factors, and kinases are identified (Yakubu et al., 2017). The Δ*prmt1* strain shows a loss of synchronous replication with abnormal daughter buds, suggesting that PRMT1 plays an essential role in centrosome dynamics during tachyzoite cell division (El Bissati et al., 2016). Briefly, epigenetic control regulates *T. gondii* stage conversion, including histone acetylation and methylation.

### Strain differences

Populations of *T. gondii* are dominated by three main lineages designated types I, II, and III in Europe and North America. At the same time, South America shows different genotypes due to more significant genetic recombination (Mukhopadhyay et al., 2020). The main genotypes in China are ToxoDB #9 (mainly type I) and #10 strains (type II) (Pan et al., 2017). The type I strains generally proliferate rapidly and are highly virulent in most inbred murine hosts, making them valuable models for studying tachyzoite growth *in vitro* and *in vivo* (Jeffers et al., 2018). The typical type I RH strain is of limited value to study the tachyzoite–bradyzoite interconversion due to the inability to form mature cysts. In contrast, type II/III strains replicate slowly and readily convert to less virulent bradyzoites in mice. They are available to study bradyzoite biology under continuous alkaline culture conditions *in vitro* (Jeffers et al., 2018). However, both the genetically modified EGS strain (type I/III) and the Obi1 strain (type II) isolated from cat feces show the ability to spontaneous form cysts without induction of bradyzoites under *in vitro* culture conditions, which are more useful for examining the developmental biology of *T. gondii* (Paredes-Santos et al., 2016; Salman et al., 2021). Based on comparative genomics of different globally distributed *T. gondii* isolates to some coccidian parasites, the study finds that amplified secreted pathogenesis determinants (SPD), including strain-specific *T. gondii* effectors (ROPs, GRAs, SRS, and TgFAMs), may influence the transmission and pathogenicity of these *T. gondii* strains (Lorenzi et al., 2016; Mukhopadhyay et al., 2020). Therefore, the roles of SPD in different genetic strains need subsequent exploration to dissect the mechanisms of tachyzoite–bradyzoite interconversion further.

## Diverse research strategies resulting in *Toxoplasma gondii* bradyzoite differentiation and maturation

### Stress-induced conversion

The bradyzoite differentiation is likely induced by stress conditions *in vitro*, and these models can overcome ethical limitations and modulate multiple experimental parameters (Cerutti et al., 2020). This subsection summarizes these factors that contribute to the differentiation process, including exogenous stresses that affect the host and parasite cellular environment and the chemical treatments that target various biological processes and pathways of *T. gondii*.

It is well known that an effective method to induce bradyzoite differentiation is to culture *T. gondii* with HFF cells under continuous alkaline culture conditions (pH 8.0–8.2, 3–4 days) or to treat parasites with an alkaline state in the absence of host cells (Skariah et al., 2010; Mayoral et al., 2020). Meanwhile, host cell heat shock (43°C) before parasite invasion (37°C) enhances bradyzoite formation (Skariah et al., 2010; Cerutti et al., 2020). Given the anti-parasitic mechanisms, IFN-γ treatment of tachyzoite-infected murine macrophages induces bradyzoite differentiation, possibly due to the nitric oxide (NO) release through stimulation of inducible nitric oxide synthase (iNOS) (Cerutti et al., 2020). Furthermore, arginine and LDL-derived cholesterol deficiencies also trigger stress-induced bradyzoite formation (Fox et al., 2004; Ihara and Nishikawa, 2014).

On the other hand, drugs targeting multiple pathways induce the conversion of tachyzoites to bradyzoites. Phosphorylated eIF2α reprograms the transcriptome and accelerates the formation and maintenance of latency (Zhang et al., 2013), so specific drugs targeting the phosphorylation/dephosphorylation processes change the frequency of bradyzoite differentiation. Tunicamycin inhibits *N*-linked glycosylation of secreted proteins and is a well-established inducer of ER stress (Das et al., 2013). Tunicamycin treatment represses the global translation initiation and protein synthesis in *T. gondii* and significantly increases *T. gondii* eIF2α phosphorylation (Joyce et al., 2013). This is consistent with the treatment of another characterized ER stress agent, the calcium ionophore A23187, but in contrast to the effect of the TgIF2K-A kinase inhibitor (GSK2606414) (Joyce et al., 2013; Augusto et al., 2018). Specific inhibitors of eIF2α dephosphorylation, salubrinal (SAL), and guanabenz (GA) similarly induce bradyzoite formation and inhibit the reactivation of bradyzoites *in vitro* (Konrad et al., 2013). In addition, the cyclic nucleotide signaling pathways play the same role in *T. gondii* stress-induced conversion (Eaton et al., 2006). Among them, forskolin, 8-(4-chlorophenylthio)-cyclic AMP (CPT-cAMP), and 1-methyl-3-isobutylxanthine (IBMX) can transiently elevate cAMP levels and induce bradyzoite differentiation (Kirkman et al., 2001; Eaton et al., 2006). Moreover, the *in vivo* study *via* intraperitoneal injection shows that guanabenz reduces brain cyst burden in chronically infected BALB/c compared with C57BL/6 mice. However, guanabenz decreases chronic inflammation in both mice (Martynowicz et al., 2019). In C57BL/6 mice, a combination of guanabenz and pyrimethamine shows synergistic efficacy on chronic toxoplasmosis, while an antagonistic effect between guanabenz and endochin-like quinolone (ELQ-334) is observed in BALB/c mice (Martynowicz et al., 2020). These drugs and compounds that target various biological processes and pathways lead to bradyzoite differentiation, highlighting the importance of evaluating anti-*T. gondii* drugs and providing an excellent opportunity for further preclinical studies to design cocktails to treat chronic toxoplasmosis.

### New *in vitro* models

Cultivating *T. gondii*-infected HFF cells using alkaline stress in the absence of CO_2_ for 3–4 days is a commonly used differentiation strategy *in vitro* (Cerutti et al., 2020). However, this condition does not allow a long-term culture of cysts, resulting in immature bradyzoite cysts. Therefore, a recent study develops a human myotube-based culture model to mature these cysts (Christiansen et al., 2022; Figure 2). In this work, KD3 human myotubes are infected with multiple strains of *T. gondii*. Nearly complete differentiation of type III VEG and intermediate maturation of type II strains in KD3 myotubes are detected by the relative number of DBA/CC2-positive versus SAG1-negative cysts under basic pH and CO_2_-deplete conditions for up to 21 days. In contrast to HFF cells, KD3 myotubes are shown to promote stage conversion and cyst maturation in multiple *T. gondii* strains under physiological conditions. The study further indicates that KD3 myotubes support cyst maturation for 35 days and that these bradyzoites *in vitro* resist pepsin, temperature stresses, and drug treatments, suggesting that this model is suitable for future drug screening against bradyzoite (Christiansen et al., 2022). Additionally, an *in vitro* model established in primary brain cells (astrocytes and neurons) reproduces spontaneous differentiation of *T. gondii* into mature bradyzoite cysts (>14 days), which is a valuable tool for studying the effect of parasite differentiation on neuron biology (Mouveaux et al., 2021; Figure 2).

These *in vitro* culture models of *T. gondii* with various cell lines are two-dimensional (2D) monolayer culture systems that are relatively easy to maintain and widely used in different experimental procedures (Ramirez-Flores et al., 2022). Studying the natural cellular structure of *T. gondii* life cycle stages is challenging due to the lack of specialized cells and limited animal models. Emerging 3D culture systems have been developed to fill the knowledge gap (Ramirez-Flores et al., 2022; Figure 2). A novel *in vitro* model about *T. gondii*-infected human brain organoids supplemented with a Matrigel matrix is successfully used to simulate cyst formation, and parasites in infected organoids retain similar pathogenicity, indicating that this physiologically relevant system helps elucidate the mechanisms of *T. gondii* stage conversion and parasite–host interactions in the brain (Seo et al., 2020).

## Discussion and outlook

The conversion of tachyzoites to bradyzoites plays a cardinal role in *T. gondii* transmission, and the reactivation of persistent bradyzoites in immunocompromised hosts may lead to severe zoonotic toxoplasmosis. Despite significant progress over the past decade in dissecting the mechanism of transition from tachyzoites to bradyzoites and vice versa, there is still much uncertainty about how host sensing and *T. gondii* self-regulation contribute to bradyzoite differentiation and reactivation. Many unknowns lead to currently ineffective treatment for underlying *T. gondii* infections.

First, tachyzoites and bradyzoites have different metabolic characteristics to meet their carbon requirements (Figure 1A). Associated metabolic enzymes in the glycolysis (ENO1) and gluconeogenesis (FBP1) pathways have high catalytic activities in bradyzoites (Kibe et al., 2005; Fleige et al., 2008). Still, it is unclear whether these characteristics are physiological determinants of bradyzoite differentiation or merely the result of the differentiation. Metabolomics studies with quantitative analysis of essential metabolites in these pathways are needed to evaluate metabolic differences at different time points during bradyzoite differentiation. These characteristics can be used to improve future bradyzoite differentiation methods *in vitro*. In particular, the regulation of polysaccharide–amylopectin metabolism by CDPK2 is essential for bradyzoites, as abnormal accumulation of amylopectin puts parasites at risk (Tomavo, 2015), so dissecting the CDPK2-dependent metabolic network helps to study its effect on the differentiation and development of bradyzoite. Under nutrient-deprived or nutrient-replete *in vitro* conditions, host metabolites that support parasite growth directly contribute to *T. gondii* stage conversion (Figure 2). These studies are limited to conventional cell types for tachyzoite proliferation *in vitro*. However, the effect of critical metabolites on bradyzoite development in muscle cells and brain cells remains unclear. Since sulfonamides and pyrimethamine, which inhibit folate synthesis, are used as effective drugs against toxoplasmosis in animals (Alday and Doggett, 2017), the next step to screen drugs targeting the central metabolic pathways of bradyzoite is of great significance for the treatment of chronic toxoplasmosis.

Additionally, despite host IFNγ-dependent defensive strategies, some tachyzoites escape immune-mediated destruction and convert to bradyzoites (Hunter and Sibley, 2012). Efficient neuronal MHC I presentation is critical for robust control of *T. gondii* (Salvioni et al., 2019). Thus, a good balance between host anti-parasitic immunity and *T. gondii* antagonism of host immunity is achieved in the chronic phase. Since a subset of neurons can clear intracellular parasites *via* immunity-regulated GTPases (Chandrasekaran et al., 2022), is there a rule to follow that some *T. gondii*-infected neurons actively harbor cysts while others do not? Single-cell RNA sequencing approaches may be necessary for revealing cell status to detect cell-to-cell differences. In immunocompromised hosts, bradyzoites can dedifferentiate into tachyzoites, depending on host CD4^+^ T cell and CD8^+^ T cell exhaustion (Bhadra et al., 2011; Hwang et al., 2016) and TgROP18-mediated phosphorylation (An et al., 2018). So, are there other models that regulate bradyzoite reactivation by host cells or by *T. gondii*? How much do they contribute to the recrudescence of toxoplasmosis in immunodeficiency individuals? In the tachyzoite–bradyzoite interconversion, unknown detail about how to maintain host immune surveillance and how *T. gondii* disrupts the balance of immune microenvironment needs to be explored.

In response to stress-induced responses, *T. gondii* effectors are involved in regulating the self-cellular environment to optimize its persistence (Figure 2). Among them, transcription activator BFD1 and bradyzoite development repressor TgAP2IX-5 are confirmed to promote or inhibit bradyzoite differentiation (Waldman et al., 2020; Khelifa et al., 2021). Consequently, both act as bridges between bradyzoite differentiation and activation, which helps to dissect the underlying molecular mechanisms of *T. gondii* stage conversion. Meanwhile, efficient proximity labeling is a powerful approach to study the global identification of *T. gondii* effectors secreted into the host cell nucleus to regulate bradyzoite differentiation (Branon et al., 2018). In addition, many GRAs are associated with tachyzoite PVM and IVN. During bradyzoite differentiation, they relocate to the cyst wall and ICN (Griffith et al., 2022), raising the possibility of a close structural correlation between vacuole and cyst. But it is unclear whether the PVM is equivalent to the cyst wall or IVN is equal to ICN, the biogenesis of *T. gondii* vacuole and cyst and their correlations need further study.

In short, the underlying molecular mechanisms of bradyzoite differentiation and reactivation remain unclear, except for a convincing causal explanation for host stress and *T. gondii* feedback. After that, studies focusing on the factors that control *T. gondii* stage conversion in an intermediate host help to understand the detailed mechanism of these processes and are promising for future drug design and vaccine development. Ultimately, better prevention and treatment can ensure that we win the tug-of-war between *T. gondii* and its hosts, reducing the public health burden of this parasite.

## Author contributions

S-YH conceived and designed the review. MP, C-CG, Y-MF, Q-WJ, and BS drafted the first version of the manuscript. All authors provided feedback on the manuscript, read, and approved the final version.
